# A Prospective Cohort Study on the Usage, Safety, and Efficacy of Delamanid in Patients With Pulmonary Multidrug-Resistant Tuberculosis in South Korea

**DOI:** 10.1093/ofid/ofaf669

**Published:** 2025-10-31

**Authors:** Jeongha Mok, Haesook Seo, Youngsoo Cho, Taehoon Lee, Sang-Ha Kim, Hye Kyeong Park, Jee Youn Oh, Tae Sun Shim

**Affiliations:** Department of Internal Medicine, Pusan National University Hospital, Pusan National University School of Medicine, Busan, Republic of Korea; Biomedical Research Institute, Pusan National University Hospital, Busan, Republic of Korea; Department of Tuberculosis, Seoul Metropolitan Seobuk Hospital, Seoul, Republic of Korea; Department of Tuberculosis, Seoul Metropolitan Seobuk Hospital, Seoul, Republic of Korea; Department of Internal Medicine, Ulsan University Hospital, University of Ulsan College of Medicine, Ulsan, Republic of Korea; Department of Internal Medicine, Yonsei University Wonju College of Medicine, Wonju, Republic of Korea; Division of Pulmonary and Critical Care Medicine, Incheon Medical Center, Incheon, Republic of Korea; Divsion of Pulmonary, Allergy, and Critical Care Medicine, Department of Internal Medicine, Korea University College of Medicine, Korea University Guro Hospital, Seoul, Republic of Korea; Department of Pulmonary and Critical Care Medicine, Asan Medical Center, University of Ulsan College of Medicine, Seoul, Republic of Korea

**Keywords:** delamanid, multidrug-resistant, tuberculosis

## Abstract

**Background:**

Delamanid has demonstrated potential in the treatment of multidrug-resistant (MDR) or rifampicin-resistant tuberculosis (TB); however, real-world data on its effectiveness and safety remain limited.

**Methods:**

This prospective cohort study enrolled patients with pulmonary MDR-TB who were treated with a delamanid-containing longer regimen under programmatic conditions in South Korea between 2017 and 2021. Data on delamanid usage, safety, and efficacy were analyzed separately.

**Results:**

In total, 147 patients were included in the usage and safety analyses (mean age, 50.7 years; 60.5% male). Adherence to delamanid was high, with a median adherence of 100.0%; 98.6% (*n* = 145) of the patients received more than 80% of the prescribed dose. Delamanid-related adverse events (AEs) occurred in 44.2% (*n* = 65) of patients, with the most common AEs being nausea (10.9%), pruritus (6.8%), and QT interval prolongation (6.1%). Serious delamanid-related AEs were reported in 4.1% (*n* = 6) of the patients, and QTcF intervals exceeding 500 ms were observed in 6.8% (*n* = 10) during treatment. For the efficacy analysis, 105 and 122 patients were included in the treatment response (at the end of delamanid treatment) and treatment outcome (at the end of MDR-TB treatment) evaluations, respectively. Among those who were culture-positive at baseline, 92.0% achieved sputum culture conversion during delamanid treatment. The overall treatment success rate was 86.9%.

**Conclusions:**

Delamanid demonstrated favorable safety and efficacy profiles for MDR-TB treatment under programmatic conditions, providing valuable and up-to-date evidence supporting its promising role in MDR-TB management.

Tuberculosis (TB) is a preventable and typically curable disease; however, it remains a significant global public health challenge. In 2023, an estimated 10.8 million individuals developed TB worldwide, and approximately 1.25 million deaths were attributed to the disease, making it one of the leading causes of mortality from a single infectious agent [1]. These persistently high incidence and mortality rates starkly contrast with the targets established by the World Health Organization (WHO) under the End TB Strategy [1]. Moreover, the global epidemic of drug-resistant TB continues to pose a major obstacle to efforts aimed at eradicating the disease. In 2023, an estimated 400 000 individuals developed multidrug-resistant (MDR) or rifampicin-resistant TB globally; however, only 40% of these cases were diagnosed and initiated on appropriate treatment, with a treatment success rate of just 68% [1].

Compared with drug-susceptible TB, the treatment of MDR-TB has traditionally required a prolonged course of second-line anti-TB drugs, which are generally less effective and associated with a higher incidence of adverse effects. Consequently, treatment outcomes have historically been poorer [2]. Additionally, resistance to key drugs used in MDR-TB treatment, such as fluoroquinolones, further complicates clinical management and limits available treatment options [3]. Nevertheless, the development and clinical introduction of new anti-TB drugs in the 2010s marked a significant turning point in the therapeutic landscape for MDR-TB.

Delamanid, a novel agent derived from the nitro-dihydro-imidazooxazole class of compounds, exerts antimycobacterial activity by inhibiting the synthesis of mycolic acid, an essential component of the mycobacterial cell wall. In preclinical studies, delamanid demonstrated potent activity against both drug-susceptible and drug-resistant strains of *Mycobacterium tuberculosis* [4, 5]. In a phase 2 clinical trial and a follow-up observational study, delamanid, when added to an optimized background regimen, resulted in higher rates of culture conversion and treatment success compared with the optimized background regimen alone in MDR-TB patients [6, 7]. Based on these findings, delamanid received conditional approval from the European Medicines Agency and was recommended by the WHO in 2014 for inclusion in an appropriate combination regimen for MDR-TB, particularly in cases where an effective regimen could not be constructed due to resistance or tolerability issues [8].

However, delamanid was not included in the subsequent large individual patient data meta-analysis, primarily due to a lack of available data at the time [9]. Furthermore, a phase 3 clinical trial demonstrated suboptimal efficacy of a delamanid-containing regimen in terms of culture conversion [10], contributing to its classification as a Group C drug in the 2019 WHO guidelines [11]. In addition, delamanid has not received approval from the United States Food and Drug Administration.

As a result, the global uptake of delamanid has been modest, and evidence regarding its effectiveness in the treatment of MDR-TB remains limited. Adverse drug reactions are a critical concern, as they can substantially influence treatment processes and patient outcomes. However, large-scale real-world data on delamanid-associated adverse events (AEs) remain limited. To address this knowledge gap, we conducted a prospective registry study to monitor and evaluate the use, safety, and efficacy of delamanid among patients with MDR-TB in South Korea under programmatic conditions.

## METHODS

### Study Design and Participants

This prospective cohort study, conducted as part of a post-marketing registry, included pulmonary MDR-TB patients at 31 hospitals across South Korea who received a delamanid-containing regimen between January 2017 and December 2021. All patients were microbiologically confirmed MDR-TB cases, diagnosed using either phenotypic or molecular drug susceptibility testing (DST). Delamanid was administered in accordance with the authorization granted by the Korean Ministry of Food and Drug Safety and the 2017 Korean TB guidelines, which align with the 2016 WHO guidelines [8, 12, 13]. Most patients met at least one of the inclusion criteria defined by the National TB Expert Review Committee for the use of new anti-TB drugs such as delamanid. These criteria included resistance or intolerance to fluoroquinolones or second-line injectable drugs; resistance or intolerance to 2 or more of the following drugs—prothionamide, cycloserine, or *P*-aminosalicylic acid; or resistance or intolerance to pyrazinamide. Patients with contraindications to delamanid, including hypersensitivity to the drug, serum albumin levels below 2.8 g/dL, concurrent use of strong cytochrome P450 inducers, or underlying conditions such as galactose intolerance, Lapp lactase deficiency, or glucose-galactose malabsorption, did not receive delamanid and were, therefore, excluded from the study.

The study protocol was reviewed and approved by the institutional review boards of all participating hospitals. Written informed consent was obtained from all study participants. The study sponsor (Korea Otsuka Pharmaceutical Co., Ltd.) was involved in the study design, data collection, and data analysis, but did not participate in data interpretation or manuscript preparation. This study was registered with ClinicalTrials.gov (NCT03470233).

### Treatment

Delamanid has been available in South Korea since September 2016. The National TB Expert Review Committee, established by the Korea Disease Control and Prevention Agency, evaluates patients for whom delamanid use is requested and provides both approval and recommendations for companion drugs. Upon approval, the cost of delamanid is fully covered by the national health insurance system. MDR-TB treatment regimens were individualized based on DST results and included at least 4 effective second-line anti-TB drugs, with or without pyrazinamide, administered for a minimum duration of 20 months [12, 13]. The use of delamanid was generally recommended during the initial 24 weeks of treatment. However, in certain cases, based on disease severity and the efficacy of the background regimen, delamanid was used beyond 24 weeks at the discretion of the attending physician, contingent upon approval from the National TB Expert Review Committee. Most patients began treatment as inpatients under directly observed therapy (DOT) and, after becoming non-infectious, continued treatment as outpatients, supported by mobile DOT provided by trained nurses.

### Data Collection and Definition

Although no strict visit schedule was predefined, most patients were followed monthly in line with routine clinical practice. Data collected included, but were not limited to, age, sex, body mass index, comorbidities, details of delamanid prescription and administration, concomitant anti-TB drugs (administered concurrently with delamanid for at least 4 weeks), laboratory test results, sputum acid-fast bacilli smear and culture results, DST results, electrocardiogram (ECG) findings, and detailed information on AEs. Most patients were advised to undergo monthly sputum examinations throughout the treatment period. Sputum cultures were performed using both liquid and solid media. ECGs were generally recommended at intervals of at least 1 month during delamanid treatment, with continued ECG monitoring in some patients following completion of delamanid therapy, at the discretion of the attending physician. The QT interval was corrected using Fridericia's formula (QTcF). Data were recorded at each site and compiled into an electronic data management system (CubeCDMS online system, version 1.0), which was specifically developed for this registry.

Regarding resistance levels of MDR-TB, our study was based on the pre-2021 classification system. Pre-extensively drug-resistant TB (pre-XDR-TB) was defined as MDR-TB with additional resistance to a fluoroquinolone or a second-line injectable drug (amikacin, kanamycin, or capreomycin), but not both. Extensively drug-resistant TB (XDR-TB) was defined as MDR-TB with additional resistance to a fluoroquinolone and at least one of these second-line injectable drugs.

### Outcomes

The delamanid usage analysis included all patients who received at least one dose of delamanid. Prescription patterns, dosages, and treatment durations were evaluated. Adherence was calculated as the ratio of the amount of delamanid actually taken to the amount prescribed. In addition, the proportion of patients who consumed more than 80% of the prescribed dose was determined.

The delamanid safety analysis also included patients who received at least one dose of delamanid. Safety data were collected from the first day of delamanid administration through 1 month following the completion of delamanid treatment. AEs were categorized by severity, including classification as serious AEs (SAEs). The occurrence of unexpected AEs (UAEs) and unexpected serious AEs (USAEs) was also assessed. All AEs were evaluated by the treating physician and the drug manufacturer to determine their causal relationship with delamanid. AEs were classified as either related or unrelated based on whether a causal link to delamanid could be excluded. All AEs were standardized using the Medical Dictionary for Regulatory Activities (MedDRA), version 24.1, and categorized according to System Organ Class and Preferred Term [14].

**Figure 1.**
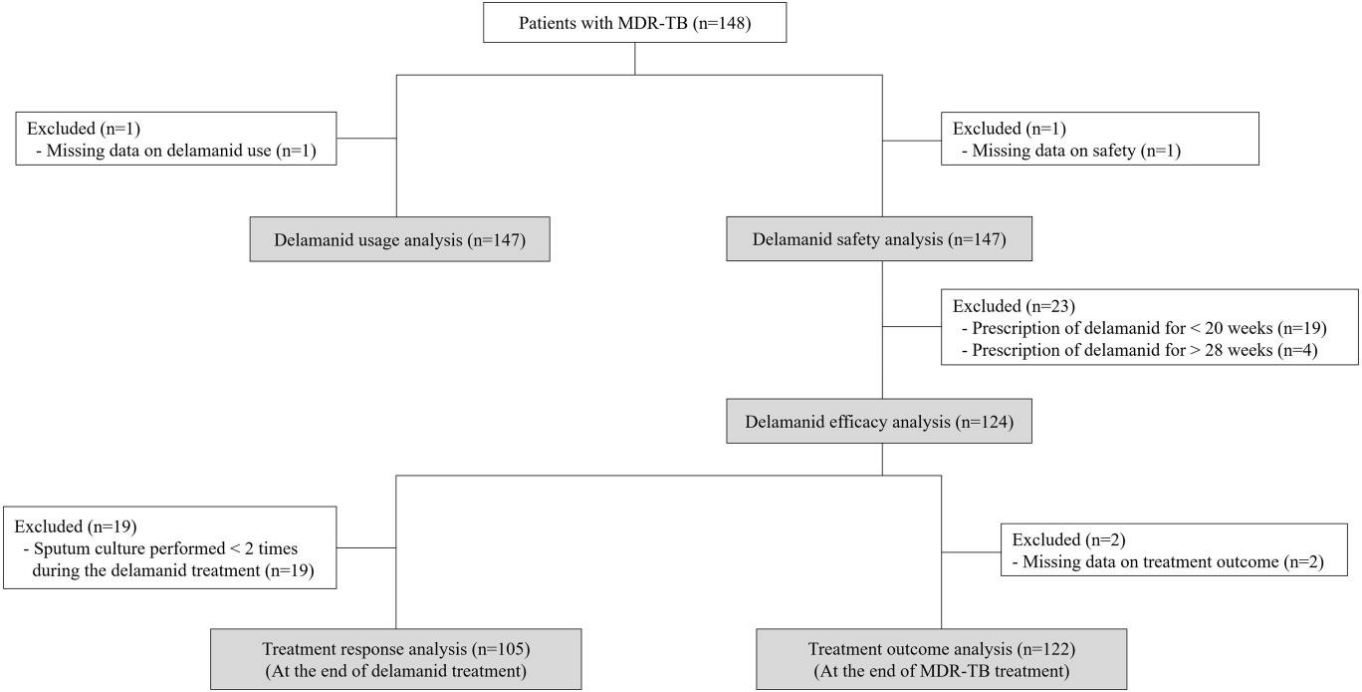
Flow chart of study participants.

The delamanid efficacy analysis included patients who received delamanid for 24 ± 4 weeks. Efficacy was assessed at 2 time points: at the end of delamanid treatment (treatment response analysis) and at the completion of MDR-TB treatment (treatment outcome analysis) (Figure 1). Within the efficacy analysis population, the treatment response analysis included patients who underwent at least 2 follow-up sputum cultures during delamanid therapy. Outcomes were classified as treatment responders or non-responders based on baseline and follow-up sputum culture results (Supplementary Appendix, Supplementary Table 1). Culture conversion was defined as 2 consecutive negative cultures collected at least 30 days apart in patients who had a positive baseline culture; the date of receipt of the first negative culture specimen was recorded as the date of culture conversion. All treatment response analyses were primarily based on cultures performed using liquid media. The treatment outcome analysis included patients from the efficacy analysis population for whom final treatment outcome data were available at the end of MDR-TB therapy. Outcomes were defined according to the 2017 Korean TB guidelines, which are consistent with the 2013 WHO definitions [12, 15] (Supplementary Appendix, Supplementary Table 2).

### Statistical Analysis

Continuous variables are presented as means with standard deviations or medians with interquartile ranges (IQRs), while categorical variables are reported as frequencies and percentages. For AEs, 95% confidence intervals were also calculated. To identify factors associated with AEs and treatment outcomes, both univariate and multivariate logistic regression analyses were performed. Variables with a *P*-value < .05 in univariate analysis, along with age and sex, were included in the multivariate model. All statistical analyses were performed using SAS software (version 9.4; SAS Institute Inc., Cary, NC, USA). A 2-tailed *P*-value of < .05 was considered statistically significant.

## RESULTS

### Baseline Participant Characteristics

In total, 148 MDR-TB patients were enrolled in the study. After excluding 1 patient with missing data, 147 patients were included in both the delamanid usage and safety analyses (Figure 1). The mean age of these 147 patients was 50.7 years, and 60.5% (*n* = 89) were male. The most common comorbidities were hypertension and diabetes mellitus, each present in 15.0% (*n* = 22) of patients. Baseline culture positivity at the time of delamanid initiation was observed in 47.6% (*n* = 70). With respect to drug resistance patterns, 43.5% (*n* = 64) of patients had MDR-TB with additional resistance to either fluoroquinolones, second-line injectable agents, or both (Pre-XDR-TB or XDR-TB) (Table 1).

**Table 1.** Baseline Characteristics of the Study Participants for Delamanid Usage and Safety Analysis^a^

Characteristic	Total Number of Patients (*n* = 147)
Sex, male	89 (60.5)
Age, yr	50.7 ± 16.7
Age group, yr	
≤ 19	3 (2.0)
20–29	14 (9.5)
30–39	23 (15.6)
40–49	31 (21.1)
50–59	31 (21.1)
≥ 60	45 (30.6)
Body mass index, kg/m^2^ (*n* = 96)	20.8 [18.9–23.2]
Comorbidity	
Hypertension	22 (15.0)
Diabetes mellitus	22 (15.0)
Chronic liver disease	8 (5.4)
Chronic kidney disease	3 (2.0)
Human immunodeficiency virus infection	1 (0.7)
Baseline *Mycobacterium tuberculosis* culture positivity	70 (47.6)
Resistance level	
MDR-TB^b^	83 (56.5)
Pre-XDR-TB^c^	44 (29.9)
XDR-TB^d^	20 (13.6)

Data are presented as mean ± standard deviation or median [interquartile range] for continuous variables and numbers (percentage) for categorical variables.

^a^The same patient (*n* = 1) was excluded from both the delamanid usage and safety analyses.

^b^Multidrug-resistant tuberculosis without additional resistance to a fluoroquinolone or a second-line injectable drug; one patient with rifampicin mono-resistant tuberculosis was included.

^c^Multidrug-resistant tuberculosis that is further resistant to either a fluoroquinolone or a second line injectable drug (amikacin, kanamycin, or capreomycin), but not both.

^d^Multidrug-resistant tuberculosis that is further resistant to a fluoroquinolone and at least one of the 3 second-line injectable drugs (amikacin, kanamycin, or capreomycin).

MDR, multidrug-resistant; pre-XDR, pre-extensively drug-resistant; TB, tuberculosis; XDR, extensively drug-resistant.

### Delamanid Usage Analysis

Among the 147 patients included in the delamanid usage analysis, 84.4% (*n* = 124) were prescribed delamanid for 24 ± 4 weeks, while 12.9% (*n* = 19) received it for less than 20 weeks, and 2.7% (*n* = 4) for more than 28 weeks (Figure 1, Table 2). Among the 19 patients prescribed delamanid for < 20 weeks, the reasons included investigator's decision (*n* = 8), AEs (*n* = 6, including 1 death), transfer out (*n* = 2), loss to follow-up (*n* = 1), withdrawal of consent (*n* = 1), and other administrative reasons (*n* = 1). The mean single prescribed dose of delamanid was 99.89 ± 1.22 mg, with an average dosing frequency of 2.00 ± 0.01 times per day. The total cumulative prescribed dose per patient was 31 488.44 ± 7254.00 mg. The median adherence to delamanid was 100.0% [IQR: 100.0–100.0], and 98.6% (*n* = 145) of the patients took more than 80% of the prescribed dose (Table 2).

**Table 2.** Delamanid Usage Analysis

	Total Number of Patients (*n* = 147)
Duration of delamanid prescription	
< 20 wks	19 (12.9)
20–28 wks	124 (84.4)
> 28 wks	4 (2.7)
Adherence to delamanid (dose taken/prescribed dose)	100.0 [100.0–100.0]
Patients with > 80% adherence to prescribed delamanid	145 (98.6)
Companion drug^a^	
Cycloserine	113 (76.9)
Protionamide	90 (61.2)
Amikacin or kanamycin	83 (56.5)
Levofloxacin or moxifloxacin	82 (55.8)
Linezolid	77 (52.4)
Para-aminosalicylic acid	43 (29.3)
Pyrazinamide	41 (27.9)
Clofazimine	40 (27.2)
Streptomycin	13 (8.8)
Bedaquiline	9 (6.1)
Number of companion drugs^a^	
≤ 2	6 (4.1)
3–4	78 (53.1)
5–7	58 (39.5)
8–10	5 (3.4)

Data are presented as medians [interquartile range] for continuous variables and numbers (percentage) for categorical variables.

^a^Anti-tuberculosis drug administered concurrently for ≥ 4 wks during the delamanid treatment period.

Among companion drugs prescribed during the delamanid treatment period, cycloserine was the most frequently used (*n* = 113, 76.9%), followed by prothionamide (*n* = 90, 61.2%) and either amikacin or kanamycin (*n* = 83, 56.5%). Linezolid was administered to 52.4% (*n* = 77) of patients. Additionally, 6.1% (*n* = 9) of patients received delamanid concurrently with bedaquiline. Most patients (53.1%, *n* = 78) received 3 to 4 companion drugs, while 39.5% (*n* = 58) received 5 to 7 drugs (Table 2).

### Delamanid Safety Analysis

Among the 147 patients included in the safety analysis, at least one AE and at least one SAE, regardless of causal relationship to delamanid, were reported in 68.0% (*n* = 100) and 15.0% (*n* = 22) of the patients, respectively (Table 3). In multivariate analysis evaluating factors associated with AE occurrence, including delamanid treatment duration, no statistically significant predictors was identified (Supplementary Appendix, Supplementary Table 3).

**Table 3.** Safety Analysis

	Total Number of Patients (*n* = 147)	
	Number of patients	95% confidence interval
At least one adverse event	100 (68.0)	59.8–75.5
At least one serious adverse event	22 (15.0)	9.6–21.8
At least one unexpected adverse event	62 (42.2)	34.1–50.6
At least one adverse event related to delamanid administration	65 (44.2)	36.0–52.6
Adverse event related to delamanid administration (number of patients ≥ 5)		
Nausea	16 (10.9)	
Pruritus	10 (6.8)	
QT interval prolongation	9 (6.1)	
Vomiting	8 (5.4)	
Dyspepsia	6 (4.1)	
Decreased appetite	6 (4.1)	
Aspartate aminotransferase increased	6 (4.1)	
Asthenia	6 (4.1)	
Alanine aminotransferase increased	5 (3.4)	
Diarrhea	5 (3.4)	
Myalgia	5 (3.4)	
Dizziness	5 (3.4)	
At least one serious adverse event related to delamanid administration	6 (4.1)	1.5–8.7
At least one unexpected adverse event related to delamanid administration	24 (16.3)	10.8–23.3
At least one unexpected serious adverse event related to delamanid administration	4 (2.7)	0.8–6.8

Data are presented as numbers (percentage).

AEs considered related to delamanid occurred in 44.2% (*n* = 65) of patients. The most frequently reported delamanid-related AE was nausea (*n* = 16, 10.9%), followed by pruritus (*n* = 10, 6.8%) and QT interval prolongation (*n* = 9, 6.1%) (Table 3). In total, 11 SAEs considered related to delamanid were reported in 4.1% (*n* = 6) of patients. These included nausea (*n* = 2), asthenia (*n* = 2), vomiting (*n* = 1), decreased appetite (*n* = 1), toxic hepatitis (*n* = 1), pulmonary embolism (*n* = 1), aggravated TB (*n* = 1), dysphemia (*n* = 1), and tremor (*n* = 1). Among these, 4 SAEs, including pulmonary embolism, aggravated TB, toxic hepatitis, and dysphemia, each occurred in different patients and were classified as USAEs. The outcome of the pulmonary embolism case could not be assessed due to withdrawal of consent during recovery. The patient with aggravated TB died during follow-up. The remaining 2 USAEs, dysphemia and toxic hepatitis, resolved without sequelae. In total, 2 patients died during the delamanid treatment period. The first patient died from aggravated TB, and a causal relationship with delamanid could not be excluded. This patient had pre-XDR-TB with fluoroquinolone resistance and died 3 months after treatment initiation. The cause of death was documented as malnutrition and general weakness secondary to TB progression. Delamanid DST was not performed in this case. The second patient died of acute respiratory acidosis resulting from aspiration pneumonia, and this death was considered unlikely to have been related to delamanid.

Between baseline and the last ECG performed during delamanid treatment, the QTcF interval increased by a mean of 14.11 ± 35.43 ms. Among the 147 patients, 6.8% (*n* = 10) experienced a QTcF interval exceeding 500 ms, and 10.2% (*n* = 15) had an increase of more than 60 ms from baseline. Among these patients, AEs considered related to delamanid were reported in 6.1% (*n* = 9).

### Delamanid Efficacy Analysis

In total, 124 patients were included in the efficacy analysis. Among them, 105 patients were evaluated in the treatment response analysis (at the end of delamanid treatment), and 122 patients were included in the treatment outcome analysis (at the end of MDR-TB treatment) (Figure 1). In the treatment response analysis, 91.4% (*n* = 96) of patients were classified as treatment responders, while 6.7% (*n* = 7) were classified as non-responders (Table 4). Among the 51 patients who were culture-positive at baseline, 92.0% (*n* = 46; excluding 1 patient for whom response assessment was not possible) achieved culture conversion during the course of delamanid treatment. The mean time to sputum culture conversion was 49.04 ± 32.18 days from delamanid therapy initiation.

**Table 4.** Efficacy Analysis

Treatment Response^a^	Total Number of Patients (*n* = 105)
Treatment responder	96 (91.4)
New converter (achieved sputum culture conversion)	46 (43.8)
Sustained converter (maintained culture negativity)	50 (47.6)
Treatment non-responder	7 (6.7)
Non-converter (failed to achieve sputum culture conversion)	4 (3.8)
Reverter (reversion^c^ to a positive sputum culture)	3 (2.9)
Cannot be assessed	2 (1.9)

Treatment outcome^b^	Total number of patients (*n* = 122)
Treatment success	106 (86.9)
Cured	92 (75.4)
Treatment completed	14 (11.5)
Treatment failed	6 (4.9)
Died	5 (4.1)
Lost to follow-up	4 (3.3)
Not evaluated	1 (0.8)

^a^At the time of delamanid treatment completion.

^b^At the time of multidrug-resistant tuberculosis treatment completion.

^c^Patients with a negative baseline culture who had at least one positive culture during the delamanid treatment period.

In the treatment outcome analysis, 86.9% (*n* = 106) of the patients achieved treatment success. The mean treatment duration among patients who achieved treatment success was 19.89 ± 5.27 months; 6 patients (4.9%) experienced treatment failure. The reasons for failure included an inability to achieve negative culture conversion during the intensive phase (*n* = 5) and permanent treatment regimen modification due to AEs (*n* = 1). In multivariate analysis, XDR-TB was significantly associated with a decreased likelihood of treatment success (Supplementary Appendix, Supplementary Table 4).

## DISCUSSION

This study evaluated the use, safety, and efficacy of delamanid in MDR-TB patients treated with a delamanid-containing longer regimen under programmatic conditions in South Korea. Delamanid was prescribed appropriately in accordance with South Korean authorization criteria, and patient adherence was high. The drug was found to be relatively safe, with no new safety signals identified. Both the treatment response during delamanid therapy and the final treatment outcomes were favorable.

At the completion of delamanid treatment, 91.4% of the patients were classified as treatment responders, defined as those who achieved sputum culture conversion or maintained culture negativity. Among patients with a positive baseline culture, the sputum culture conversion rate was 92.0%, which exceeded the rates reported in a phase 3 clinical trial and a large observational study [10, 16], and was comparable to the 91.7% reported in a previous study conducted in South Korea [17]. The overall treatment success rate was 86.9%, surpassing the 76.5% reported in a phase 3 trial and the 80.9% found in a recent meta-analysis [10, 18]. By contrast, national data from South Korea during the pre-delamanid era (2011–2014) indicated an overall treatment success rate of 65.7% among MDR-TB patients, and only 53.1% among those with XDR-TB [19]. Considering that nearly half of the patients in our cohort had resistance to fluoroquinolones or second-line injectable agents, this significant improvement is encouraging. While part of this improvement may be attributable to overall advancements in MDR-TB management, such as the implementation of rapid molecular DST, increased use of potent drugs like linezolid, and systematic patient-centered care, the clinical introduction of new anti-TB agents, including delamanid, likely played a central role [20, 21]. A previous study from South Korea similarly reported that the introduction of new drugs, including delamanid, significantly contributed to improved treatment success [21].

High adherence to delamanid was observed, and no new safety concerns emerged during the study period. Most AEs, including nausea, vomiting, pruritus, and QT interval prolongation, were anticipated and consistent with previous studies [10, 18, 22, 23]. The incidence of delamanid-related SAEs was low. Only 6 patients (4.1%) discontinued delamanid prematurely due to AEs, a rate comparable to the 6% reported in a European post-authorization safety study [23]. A global surveillance report also identified delamanid as one of the safest anti-TB medications [22]. QT interval prolongation is a risk associated with several MDR-TB drugs, including fluoroquinolones, bedaquiline, delamanid, and clofazimine. Despite the frequent co-administration of other QT-prolonging agents in our cohort (fluoroquinolones in 56% and clofazimine in 27%), only 6.8% of the patients had a QTcF interval ≥ 500 ms, which aligns with previous findings [10, 17, 18, 23]. Reports of ventricular arrhythmias or sudden cardiac death linked to delamanid remain extremely rare, and no such events occurred in our study. These findings imply that delamanid may present a lower cardiotoxic risk than initially anticipated.

Bedaquiline, another novel anti-TB drug, was introduced into clinical use earlier than delamanid and has become a cornerstone in both longer and shorter MDR-TB regimens based on extensive clinical experience. In contrast, delamanid's role has remained relatively limited. However, to date, direct comparative data between these 2 drugs are still limited. A previous study in South Korea comparing bedaquiline- and delamanid-containing longer regimens (*n* = 119 and = 141, respectively) found no significant difference in treatment success (75.6% for bedaquiline vs 82.3% for delamanid) and reported similar safety profiles [17]. Another cohort study, involving patients with similar characteristics to those in our study, reported a treatment success rate of only 56.3% with a bedaquiline-containing longer regimen, much lower than the 91.7% observed in our cohort [24]. Furthermore, recent meta-analyses have reported comparable success rates for bedaquiline and delamanid (74.7% vs 80.9%) [18, 25]. These findings highlight the need to re-evaluate the role of delamanid in MDR-TB treatment as clinical experience continues to accumulate.

Reflecting domestic clinical experience and emerging evidence, the revised Korean tuberculosis guidelines, effective from 2024, now classify delamanid as a Group A drug alongside bedaquiline for use in longer MDR-TB regimens, thereby permitting its use under the same conditions as bedaquiline [26]. Additionally, a 9-month shorter regimen incorporating delamanid, the “MDR-END” regimen (levofloxacin, delamanid, linezolid, and pyrazinamide), is now recommended in South Korea for fluoroquinolone-susceptible MDR-TB, in parallel with WHO-endorsed regimens, such as BPaLM [26, 27]. The WHO has also recently updated its guidelines to include several new shorter regimens incorporating delamanid [28]. The global expansion of delamanid use is expected to enhance understanding of its clinical value. Moreover, given increasing concerns about bedaquiline resistance [29], delamanid may assume a critical role in bedaquiline-sparing regimens.

Nonetheless, several challenges must be overcome to expand further the clinical use of delamanid. Chief among them is the lack of DST to guide its use. Globally, the development and implementation of phenotypic DST for delamanid have been slow, and commercially available rapid molecular DSTs are scarce. In the absence of DST-guided treatment, the emergence of acquired resistance is almost inevitable. Therefore, the development and scale-up of rapid, reliable, and reproducible DST methods are essential.

This study had several limitations. First, DST for delamanid was not fully implemented in South Korea during the study period, resulting in limited availability of DST data. In this study, only 6 patients underwent minimum inhibitory concentration testing at baseline or during delamanid treatment, and none demonstrated resistance. Second, pediatric patients were not included in the analysis. However, based on recent evidence, delamanid is no longer age-restricted in the treatment of MDR-TB. Third, our study did not include data on the presence of cavities or smear results, which are important indicators of bacillary burden and disease severity in TB. Fourth, since this was not a clinical trial with a standardized protocol and fixed timelines, certain outcomes, such as the time to sputum culture conversion, may lack precision. Finally, the efficacy analyses were restricted to patients who received delamanid for 24 ± 4 weeks, in accordance with regulatory approval, which may have led to an overestimation of treatment response or outcomes.

Despite these limitations, our study provides prospectively and systematically collected data from a large cohort of MDR-TB patients treated under real-world programmatic conditions. The findings offer valuable evidence on the efficacy, effectiveness, and safety of delamanid, supporting its potential role in MDR-TB treatment. Further large-scale data collection and clinical studies are warranted to strengthen the evidence base and more clearly define the role of delamanid in the management of MDR-TB.

## Supplementary Material

ofaf669_Supplementary_Data

## Supplementary Data

Supplementary materials are available at *Open Forum Infectious Diseases* online. Consisting of data provided by the authors to benefit the reader, the posted materials are not copyedited and are the sole responsibility of the authors, so questions or comments should be addressed to the corresponding author.
