# Supplementary material for: A Prospective Cohort Study on the Usage, Safety, and Efficacy of Delamanid in Patients With Pulmonary Multidrug-Resistant Tuberculosis in South Korea
Source: Open Forum Infect Dis. 2025 Oct 31;12(11):ofaf669. doi: 10.1093/ofid/ofaf669 (PMC12628499; doi:10.1093/ofid/ofaf669)
Supplement: ofaf669_Supplementary_Data [file ofaf669_supplementary_data.docx]

**Supplementary Material**

Table S1. Definitions for treatment response analysis.

| Treatment responder | New converter | Patients with a positive baseline culture who achieved sputum culture conversion^a^ during the delamanid treatment period. |
| --- | --- | --- |
|  | Sustained converter | Patients with a negative baseline culture who maintained culture negativity throughout the delamanid treatment period. |
| Treatment non-responder | Non-converter | Patients with a positive baseline culture who did not achieve sputum culture conversion^a^ during the delamanid treatment period. |
|  | Reverter | Patients with a negative baseline culture who had at least one positive culture during the delamanid treatment period. |

^a^Two consecutive negative cultures, taken at least 30 days apart, in a patient who had a positive culture result at baseline.

Table S2. Definitions for treatment outcome analysis.

| Outcome | Definition |
| --- | --- |
| Cured | Treatment completed as recommended by the national policy without evidence of failure and three or more consecutive cultures taken at least 30 days apart were negative after the intensive phase^a^. |
| Treatment completed | Treatment completed as recommended by the national policy without evidence of failure, but no record that three or more consecutive cultures, taken at least 30 days apart, were negative after the intensive phase^a^. |
| Treatment failed | Treatment terminated or need for permanent regimen change of at least two anti-tuberculosis drugs because of any of the following:  - lack of conversion^b^ by the end of the intensive phase^a^  - bacteriological reversion^c^ in the continuation phase after conversion^b^ to negative  - evidence of additional acquired resistance to fluoroquinolones or second-line injectable drugs  - adverse drug reactions |
| Died | A patient who died due to any cause during the course of treatment. |
| Lost to follow-up | A patient whose treatment was interrupted for two or more consecutive months. |
| Not evaluated | A patient for whom no treatment outcome was assigned (including cases that were “transferred out” to another treatment unit and whose treatment outcome was unknown). |
| Treatment success | The sum of cured and treatment completed |

^a^8-month cut-off was applied.

^b^Conversion (to negative): Culture was considered converted to negative when two consecutive cultures, taken at least 30 days apart, were found to be negative. In such cases, the specimen collection date for the first negative culture was considered the date of conversion.

^c^Reversion (to positive): Culture was considered to have reverted to positive when, after an initial conversion, two consecutive cultures, taken at least 30 days apart, were found to be positive. For defining “treatment failed”, reversion was considered only when it occurred in the continuation phase.

Table S3. Factors associated with adverse events.

|  | Univariate^a^ | | | Multivariate^b^ | | |
| --- | --- | --- | --- | --- | --- | --- |
|  | OR | 95% CI | *P*-value | OR | 95% CI | *P*-value |
| Age group, y |  |  |  |  |  |  |
| ≤ 19 | Reference | | | Reference | | |
| 20–29 | 0.247 | 0.007–8.941 | 0.445 | 0.297 | 0.007–11.803 | 0.519 |
| 30–39 | 0.385 | 0.011–13.297 | 0.597 | 0.641 | 0.017–24.354 | 0.811 |
| 40–49 | 0.395 | 0.012–13.253 | 0.604 | 0.457 | 0.013–16.388 | 0.668 |
| 50–59 | 0.255 | 0.008–8.428 | 0.444 | 0.269 | 0.008–9.587 | 0.472 |
| ≥ 60 | 0.233 | 0.007–7.507 | 0.411 | 0.348 | 0.010–12.240 | 0.561 |
| Sex, male | 1.065 | 0.526–2.159 | 0.861 | 1.158 | 0.521–2.577 | 0.719 |
| BMI < 18.5, kg/m^2^ (n = 96) | 0.635 | 0.234–1.726 | 0.374 |  |  |  |
| Hypertension, yes | 1.619 | 0.568–4.616 | 0.367 |  |  |  |
| Diabetes mellitus, yes | 1.247 | 0.459–3.389 | 0.666 |  |  |  |
| Chronic liver disease, yes | 8.721 | 0.415–183.066 | 0.163 |  |  |  |
| Chronic kidney disease, yes | 3.408 | 0.109–106.132 | 0.485 |  |  |  |
| Resistance level |  |  |  |  |  |  |
| MDR-TB^c^ | Reference | | | Reference | | |
| Pre-XDR-TB^d^ | 2.735 | 1.175–6.367 | 0.020 | 1.789 | 0.697–4.591 | 0.227 |
| XDR-TB^e^ | 3.660 | 1.044–12.824 | 0.043 | 2.304 | 0.605–8.768 | 0.221 |
| Duration of delamanid prescription |  |  |  |  |  |  |
| 20–28 weeks | Reference | | | Reference | | |
| < 20 weeks | 2.516 | 0.728–8.693 | 0.145 |  |  |  |
| > 28 weeks | 1.245 | 0.142–10.904 | 0.843 |  |  |  |
| Companion drug^f^ |  |  |  |  |  |  |
| Cycloserine | 0.731 | 0.312–1.710 | 0.469 |  |  |  |
| Levofloxacin or moxifloxacin | 0.416 | 0.200–0.867 | 0.019 | 0.734 | 0.303–1.780 | 0.494 |
| Amikacin or kanamycin | 1.072 | 0.534–2.153 | 0.844 |  |  |  |
| Linezolid | 2.286 | 1.126–4.642 | 0.022 | 1.300 | 0.520–3.250 | 0.575 |
| Clofazimine | 2.680 | 1.098–6.540 | 0.030 | 1.869 | 0.688–5.075 | 0.220 |
| Streptomycin | 0.710 | 0.220–2.290 | 0.566 |  |  |  |
| Bedaquiline | 2.849 | 0.442–18.367 | 0.271 |  |  |  |
| Prothionamide | 1.111 | 0.547–2.253 | 0.771 |  |  |  |
| Para-aminosalicylic acid | 0.953 | 0.447–2.034 | 0.901 |  |  |  |
| Pyrazinamide | 1.172 | 0.536–2.561 | 0.691 |  |  |  |

^a^147 patients from the safety analysis population were included (except for body mass index, for which, n = 96).

^b^96 patients were included, excluding 51 with missing data on body mass index.

^c^Multidrug-resistant tuberculosis without additional resistance to a fluoroquinolone or a second-line injectable drug; one patient with rifampicin mono-resistant tuberculosis was included.

^d^Multidrug-resistant tuberculosis that is further resistant to either a fluoroquinolone or a second line injectable drug (amikacin, kanamycin, or capreomycin), but not both.

^e^Multidrug-resistant tuberculosis that is further resistant to a fluoroquinolone and at least one of the three second-line injectable drugs (amikacin, kanamycin, or capreomycin).

^f^Anti-tuberculosis drug administered concurrently for ≥ 4 weeks during the delamanid treatment period.

BMI, body mass index; CI, confidence interval; MDR, multidrug-resistant; OR, odds ratio; pre-XDR, pre-extensively drug-resistant; TB, tuberculosis; XDR, extensively drug-resistant.

Table S4. Factors associated with treatment success.

|  | Univariate^a^ | | | Multivariate^b^ | | |
| --- | --- | --- | --- | --- | --- | --- |
|  | OR | 95% CI | *P*-value | OR | 95% CI | *P*-value |
| Age group, y |  |  |  |  |  |  |
| ≤ 19 | Reference | | | Reference | | |
| 20–29 | 3.569 | 0.039–325.570 | 0.581 | 5.518 | 0.058–524.227 | 0.462 |
| 30–39 | 0.555 | 0.015–19.975 | 0.748 | 0.836 | 0.023–30.653 | 0.923 |
| 40–49 | 0.506 | 0.014–17.729 | 0.707 | 0.912 | 0.025–32.710 | 0.960 |
| 50–59 | 1.399 | 0.036–54.936 | 0.858 | 2.955 | 0.071–122.382 | 0.568 |
| ≥ 60 | 0.818 | 0.024–28.280 | 0.911 | 1.207 | 0.034–42.371 | 0.918 |
| Sex, male | 0.904 | 0.313–2.611 | 0.852 | 1.008 | 0.339–2.995 | 0.989 |
| BMI < 18.5, kg/m^2^ (n = 78) | 0.817 | 0.205–3.250 | 0.774 |  |  |  |
| Hypertension, yes | 0.703 | 0.189–2.620 | 0.600 |  |  |  |
| Diabetes mellitus, yes | 2.303 | 0.386–13.742 | 0.360 |  |  |  |
| Chronic liver disease, yes | 0.314 | 0.058–1.705 | 0.180 |  |  |  |
| Chronic kidney disease, yes | 0.147 | 0.009–2.471 | 0.183 |  |  |  |
| Resistance level |  |  |  |  |  |  |
| MDR-TB^c^ | Reference | | | Reference | | |
| Pre-XDR-TB^d^ | 1.822 | 0.411–8.084 | 0.430 | 1.659 | 0.408–6.751 | 0.479 |
| XDR-TB^e^ | 0.241 | 0.071–0.819 | 0.023 | 0.228 | 0.062–0.833 | 0.025 |
| Companion drug^f^ |  |  |  |  |  |  |
| Cycloserine | 0.728 | 0.170–3.115 | 0.668 |  |  |  |
| Levofloxacin or moxifloxacin | 1.460 | 0.519–4.108 | 0.474 |  |  |  |
| Amikacin or kanamycin | 2.183 | 0.757–6.297 | 0.148 |  |  |  |
| Linezolid | 0.883 | 0.313–2.495 | 0.815 |  |  |  |
| Clofazimine | 1.538 | 0.434–5.446 | 0.505 |  |  |  |
| Streptomycin | 0.420 | 0.106–1.667 | 0.217 |  |  |  |
| Bedaquiline | 0.453 | 0.056–3.681 | 0.459 |  |  |  |
| Prothionamide | 0.886 | 0.294–2.668 | 0.830 |  |  |  |
| Para-aminosalicylic acid | 0.482 | 0.167–1.389 | 0.177 |  |  |  |
| Pyrazinamide | 0.834 | 0.274–2.535 | 0.749 |  |  |  |

^a^122 patients from the treatment outcome analysis population were included (except for body mass index, for which, n = 78).

^b^78 patients were included, excluding 44 patients with missing data on body mass index.

^c^Multidrug-resistant tuberculosis without additional resistance to a fluoroquinolone or a second-line injectable drug; one patient with rifampicin mono-resistant tuberculosis was included.

^d^Multidrug-resistant tuberculosis that is further resistant to either a fluoroquinolone or a second line injectable drug (amikacin, kanamycin, or capreomycin), but not both.

^e^Multidrug-resistant tuberculosis that is further resistant to any fluoroquinolone and at least one of the three second-line injectable drugs (amikacin, kanamycin, or capreomycin).

^f^Anti-tuberculosis drug administered concurrently for ≥ 4 weeks during the delamanid treatment period.

BMI, body mass index; CI, confidence interval; MDR, multidrug-resistant; OR, odds ratio; pre-XDR, pre-extensively drug-resistant; TB, tuberculosis; XDR, extensively drug-resistant.
